# Error model of geomagnetic-field measurement and extended Kalman-filter based compensation method

**DOI:** 10.1371/journal.pone.0173962

**Published:** 2017-04-26

**Authors:** Zhilei Ge, Suyun Liu, Guopeng Li, Yan Huang, Yanni Wang

**Affiliations:** School of Astronautics, Northwestern Polytechnical University, Xi'an, Shaan xi Province, People’s Republic of China; Chongqing University, CHINA

## Abstract

The real-time accurate measurement of the geomagnetic-field is the foundation to achieving high-precision geomagnetic navigation. The existing geomagnetic-field measurement models are essentially simplified models that cannot accurately describe the sources of measurement error. This paper, on the basis of systematically analyzing the source of geomagnetic-field measurement error, built a complete measurement model, into which the previously unconsidered geomagnetic daily variation field was introduced. This paper proposed an extended Kalman-filter based compensation method, which allows a large amount of measurement data to be used in estimating parameters to obtain the optimal solution in the sense of statistics. The experiment results showed that the compensated strength of the geomagnetic field remained close to the real value and the measurement error was basically controlled within 5nT. In addition, this compensation method has strong applicability due to its easy data collection and ability to remove the dependence on a high-precision measurement instrument.

## 1. Introduction

The geomagnetic-field is an inherent physical field of the earth. For navigation and orientation, the geomagnetic-field has several advantages. It is strongly self-contained and has anti-interference and all-weather features. Due to its small size, geomagnetic navigation equipment has low energy consumption and low cost. For these reasons, geomagnetic navigation is increasingly attracting the attention of domestic and foreign scholar and has become a highlight in the field of navigation around the world [1]. The accurate acquisition of geomagnetic information is the precondition for geomagnetic navigation and the basis for achieving high-precision geomagnetic navigation at the same time. Currently, a navigation carrier mostly measures magnetic information of the space via a strap-down magnetic sensor. However, as the spectral range of the geomagnetic field is very wide, the measurement of the geomagnetic field is subject to interference, causing the output of the magnetic sensor to not only contain the geomagnetic information needed for navigation and orientation but also contain various interferential information. To promote the accuracy of geomagnetic navigation and meet the requirement of high-precision geomagnetic navigation for highly accurate geomagnetic-field information, the output information of the magnetic sensor must be corrected [2–3].

By referring to the research findings of domestic and foreign scholars, this paper divided the sources of the measurement error of magnetic sensors into manufacturing error, installation error and interference from the external electromagnetic environment [4–13]. The study of an existing magnetic-interference model found that the simplified model cannot clearly describe the sources of measurement error of magnetic sensors. A 12-parameter error model including manufacturing error and installation error was built [4]. An error model including manufacturing error and interference from the external electromagnetic environment was built [5]. The error model built in [6–8] only mentioned the sensor’s manufacturing error. The error model built in [9–14] only considered the interference of the external electromagnetic environment. It is necessary to note that these models do not address daily variations of the geomagnetic-field in the study of the interference of the external electromagnetic environment. As the major component of magnetic-field variation in quiet periods of geomagnetism, the accurate measurement of the geomagnetic daily variation field has a non-negligible effect on the accurate measurement of the geomagnetic-field [15]. To ensure the accuracy of measurement data, the model must be corrected based on the geomagnetic daily variation field.

Regarding compensation for magnetic-field interference, the existing methods include the multi-pose method, ellipse-fitting method, ellipsoid-fitting method, neural network method, deviation-compensation method, Tolles-Lawson equation-based magnetic-measurement compensation method and Kalman filtering. The “ellipse assumption method of two-dimensional magnetic field measuring track” was proposed [4]. The core idea of this method is to convert the problem of compensation of magnetic measurements to a problem of parameter estimation. However, two estimation processes contained in this algorithm were non-linear parameter estimations and such estimation processes are extremely complicated and involve large amounts of calculations. In the process of estimating the ellipse parameters in the first step, since the ellipticity of the conic section cannot be ensured, the ordinary least-square method is not applicable and this further intensifies the complexity of algorithm. In addition, this method can only solve for 9 of the 12 parameters of the constructed model; the estimation of the remainder of the parameters requires the help of accelerometer and reference information provided by GPS, which intensifies the complexity of the algorithm structure and increases the number of calculations.

A method based on ellipse fitting was proposed in [11–12] to use a measured value to fit an ellipse and find ellipse parameters, and obtain the error model’s parameters in accordance with the relation between the ellipse parameters and error model. The algorithm of this method is rather complicated and it strictly requires the carrier to move only in the horizontal plane during data acquisition, therefore this method is only applicable to two-dimensional space and is greatly limited in practical application.

References [6, 9, 16–19] put forward ellipsoid fitting algorithm, which calculates ellipse parameters with least-square method or iterative algorithm and indirectly performs error compensation based on the characteristic that the track of the geomagnetic field subjected to various interferences in a fixed space is ellipsoid. Although the ellipsoid fitting algorithm is able to obtain an ideal compensation effect, there is such a problem that iterative algorithm doesn't only require a large number of calculations but also relatively accurate initial conditions, and there is likely to be the problem of matrix singularity in the solving process of least-square method, making it unable to obtain the correct solution.

References [18–19] both proposed to use neural network algorithm for the compensation of carrier’s magnetic field: reference [18] applied neural network algorithm in the parameter estimation of ellipsoidal model, and reference [19] compared neural network algorithm with ellipsoid fitting algorithm in terms of the result of the error compensation for magnetic measurement. The final results of both studies revealed that the neural network algorithm was able to achieve a good effect with respect to compensation accuracy, but its effect was not better than that of the ellipsoid fitting algorithm. Furthermore, in consideration of the long time it takes in model training, the application of this method is greatly limited.

The compensation effects of ellipse fitting algorithm, ellipsoid fitting algorithm and neural network algorithm are all sensitive to the measuring accuracy and noise of sensor, so additional measures are required to solve this problem; for example, reference [18] adopted wavelet de-noising method to eliminate the effect of noise, which undoubtedly increased the workload of compensation. As a recursive algorithm, Kalman-filter algorithm is able to achieve online operation and has a certain adaptability to the errors in initial states and a good ability to suppress noise; therefore it has been extensively used in the estimation and calibration of various models' parameters [20–25]. Although the extended Kalman-filter algorithm has been applied in geomagnetic navigation technology to a certain degree, the applications are mostly focused on the fusion of navigation information [26–27], and there lack the reports on error compensation for magnetic measurement.

During data acquisition, ellipse fitting method requires the carrier to move only in a two-dimensional space [11]; multi-pose method requires rotating a specially made hexahedral apparatus to obtain 12 or 24 different poses [28]; despite the simplicity of data acquisition, ellipsoid fitting method is unable to reach a stable solution in the end because constraint matrix singularity will be caused when the ellipsoid where the data acquired are approaches a sphere [16–17]. By contrast, the method proposed in the paper requires the sensor only to rotate in a three-dimensional space during data acquisition, and there are no additional constraints, so it has better adaptability.

On the basis of study done by others, this paper improved the existing magnetic-sensor measurement-error model, introduced the geomagnetic daily variation field and built a new model that includes the magnetic sensor’s manufacturing error, installation error and the interference of the external electromagnetic environment. The new model is able to indicate the source of the measurement error. With respect to the estimation of the model parameter, an extended Kalman-filter with the model parameter as the state variable and the magnetic sensor’s output quantity as the observation quantity was designed according to the model’s characteristics. The new filter can fully make use of all measured data to resolve the problem of the poor utilization rate of the measured data. In addition, with the increase in the amount of measured data, the estimated value of the model parameter will well converge to the real value to obtain the optimal solution in the sense of statistics. In addition, due to the Kalman filter’s good capability to constrain noise, this method can effectively prevent strange solutions in the process of seeking the solution and eventually realize high-precision compensation of measured data.

## 2. Magnetic sensor’s measurement-error model

This paper will proceed from the magnetic sensor’s manufacturing error, installation error and interference of the external electromagnetic environment to study each of these three factors in detail and build their measurement-error models. Finally, a complete model containing the above three factors will be built.

### 2.1. Manufacturing-error model

**Fig 1:**
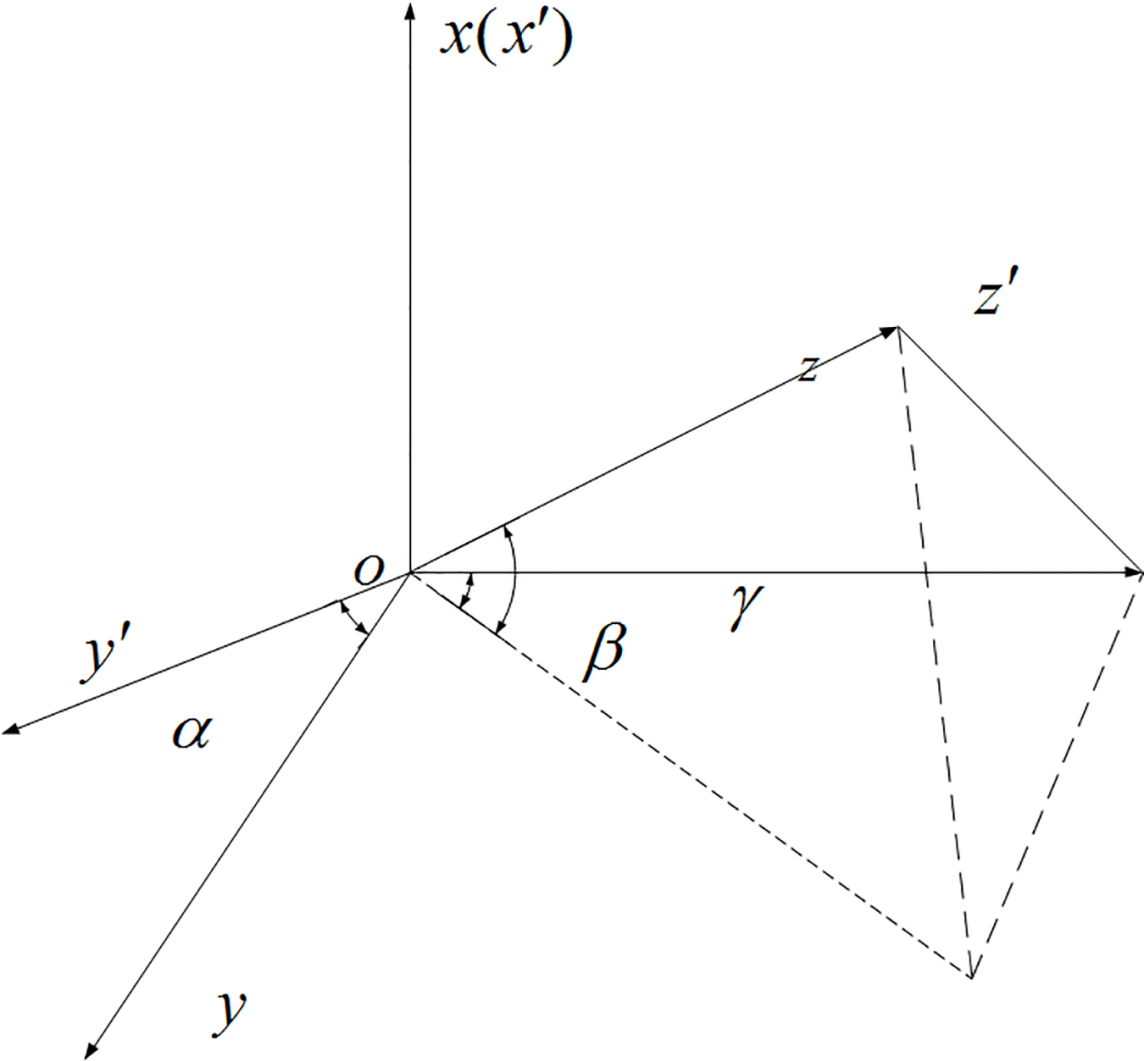
Magnetic sensor’s ideal and real coordinates.

Manufacturing error, caused by the level of production technology, material properties and many other factors that make the sensor unable to reach the ideal working conditions, mainly includes sensitivity error, zero error and non-orthogonal error. Sensitivity error is the result of the different sensitivities of the magnetic sensor’s three measuring axes and can be represented using a diagonal matrix. Zero error occurs because the zero points of the geomagnetic sensor, analog circuit and analog digital converter are not zero and can be represented using a fixed vector form. Non-orthogonal error occurs because the actual directions of the magnetic sensor’s three measuring axes are not completely orthogonal and its principle is shown in Fig 1 *o*–*xyz* represents the ideal coordinates of the geomagnetic sensor’s measuring axes. This set of coordinates is an orthogonal one, while the actual coordinates of the geomagnetic sensor’s measuring axes, *o*–*x*′*y*′*z*′, are non-orthogonal. The origin *o* of the coordinates is the center of the geomagnetic sensor. The *ox*-axis coincides with the *ox*′-axis; the *o*–*xy* plane coincides with the *o*–*x*′*y*′ plane; and the included angle of the *oy*-axis and *oy*′-axis is *α*. The included angle between the projection of the *o*–*z*′-axis on the *o*–*yz* plane and the *o*–*z*-axis is *β*, and the included angle between the *o*–*z*′-axis and its projection is *γ* [5–6].

Assuming that the magnetic sensor rotates in a certain fixed area and under the condition of only considering the effect of its manufacturing error, the relation between the magnetic output and the real magnetic field of the area is [7–8]

$$H_{m}=SPH_{i}+b_{o},$$ (1)

where *H*_*m*_ represents the output of the magnetic sensor’s three axes and *H*_*i*_ represents the magnetic-field vector in the sensor’s coordinates.

$$S=\left[\begin{array}{ccc} 1+s_{x} & 0 & 0\\ 0 & 1+s_{y} & 0\\ 0 & 0 & 1+s_{z} \end{array}\right]\;\mathrm{and}\;P=\left[\begin{array}{ccc} 1 & 0 & 0\\ \sin\alpha & \cos\alpha & 0\\ \sin\gamma & \sin\beta \cos\gamma & \cos\beta \cos\gamma \end{array}\right]$$

respectively, represent the sensitivity matrix and non-orthogonal matrix. *s*_*x*_ and *s*_*y*_ and *s*_*z*_ respectively, represent the sensitivity coefficients of the geomagnetic sensor’s three axes and are constants. *b*_*o*_ represents the zero error: *b*_*o*_ = [*b*_*ox*_ *b*_*oy*_ *b*_*oz*_]^T^, and it can be regarded as a fixed vector.

### 2.2. Magnetic sensor’s installation-error model

**Fig 2:**
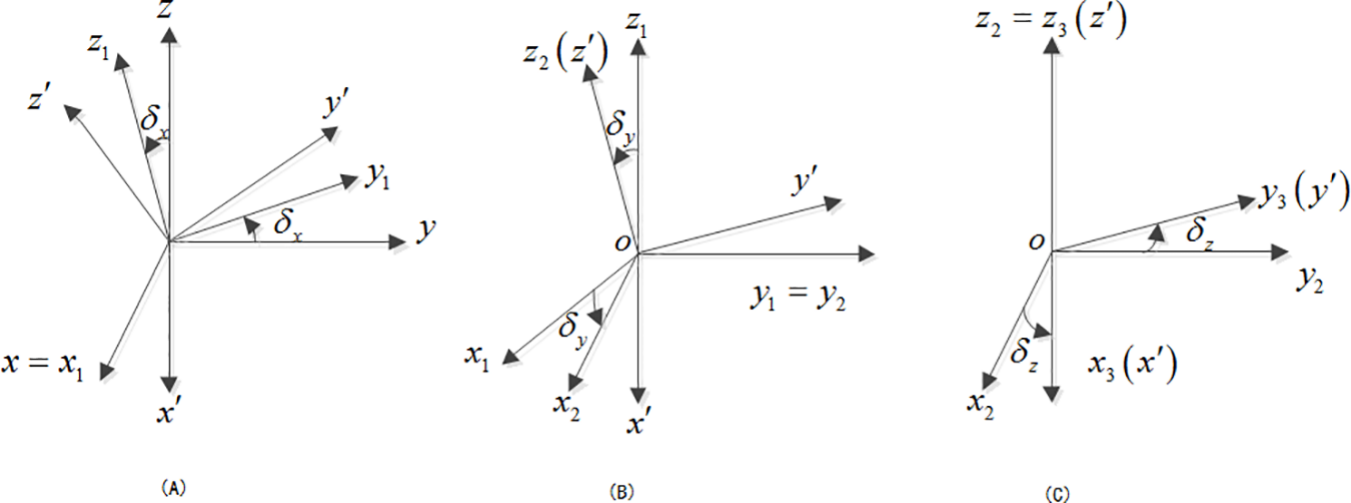
Transformation between magnetic sensor’s coordinates and carrier’s coordinates. (A) Rotate around x-axis. (B) Rotate around y-axis. (C) Rotate around z-axis.

The difference between the actual installation position and the ideal installation position is the main cause of the magnetic sensor’s installation error. The transformation process between the two coordinates is shown in Fig 2: *o*–*x*′*y*′*z*′ represents the carrier’s coordinates and *o*–*xyz* represents the magnetic sensor’s coordinates. The three transformation processes completely reflect the transformation from the magnetic sensor’s coordinates to the carrier’s coordinates [4–5].

Under the condition of only considering the effect of the installation error, the transformation of the geomagnetic field from the magnetic sensor’s coordinates to the carrier’s coordinates is

$$H_{m}=E_{p}H_{i},$$ (2)

where *H*_*m*_ represents the magnetic-field vector in the carrier’s coordinates, *H*_*i*_ represents the magnetic-field vector in the sensor’s coordinates and *E*_*p*_ represents the installation-error matrix.

$$E_{p}=\left[\begin{array}{ccc} 1 & 0 & 0\\ 0 & \cos\delta_{x} & \sin\delta_{x}\\ 0 & -\sin\delta_{x} & \cos\delta_{x} \end{array}\right]\left[\begin{array}{ccc} \cos\delta_{y} & 0 & \sin\delta_{y}\\ 0 & 1 & 0\\ -\sin\delta_{y} & 0 & \cos\delta_{y} \end{array}\right]\left[\begin{array}{ccc} \cos\delta_{z} & \sin\delta_{z} & 0\\ -\sin\delta_{z} & \cos\delta_{z} & 0\\ 0 & 0 & 1 \end{array}\right],$$

For the same carrier, the strap-down magnetic sensor will not be adjusted in position once it is fixed, therefore, the installation-error matrix *E*_*p*_ will not change under normal conditions.

### 2.3. Interference-error model of the external electromagnetic environment

The magnetic sensor’s measurement of the geomagnetic-field is likely to be subject to interference from the surrounding electromagnetic environment, which is determined by the characteristic wide spectral range of the geomagnetic-field. The interference from the external electromagnetic environment includes hard magnetic interference, soft magnetic interference, random magnetic-field interference and geomagnetic daily variation interference. Hard magnetic interference refers to the interfering magnetic field that is formed as the hard magnetic material inside the carrier is magnetized by the external magnetic field. As hard magnetic material has high coercive force and a remanence value that can remain unchanged over a long period, the interfering field formed by hard magnetic material remains unchanged in the carrier’s fixed coordinates and it can be regarded as a fixed vector. Soft magnetic interference refers to the induced magnetic field that is formed as soft magnetic material inside the carrier is magnetized by the external magnetic field. Different from hard magnetic interference, soft magnetic interference will vary with the variation of the external magnetic field, and the carrier’s soft magnetic interfering field can be regarded as the summation of a number of magnetized magnetic dipoles and magnetic-dipole moments [12]. The induced magnetic field is described in detail as follows. Assume that *M*_*x*_,*M*_*y*_,*M*_*z*_ are the three components of the induced magnetic moment produced by *H*_*ix*_,*H*_*iy*_,*H*_*iz*_, respectively, that magnetize soft iron in the sensor’s coordinates. The induced magnetic moment in the three axial directions in the carrier’s coordinates is

$$\{\begin{array}{c} M_{x}=K_{x}H_{ix};\\ M_{y}=K_{y}H_{iy};\\ M_{z}=K_{z}H_{iz}. \end{array},$$ (3)

where *K*_*x*_,*K*_*y*_,*K*_*z*_ are the magnetized coefficients, which are non-dimensional constants.

**Fig 3:**
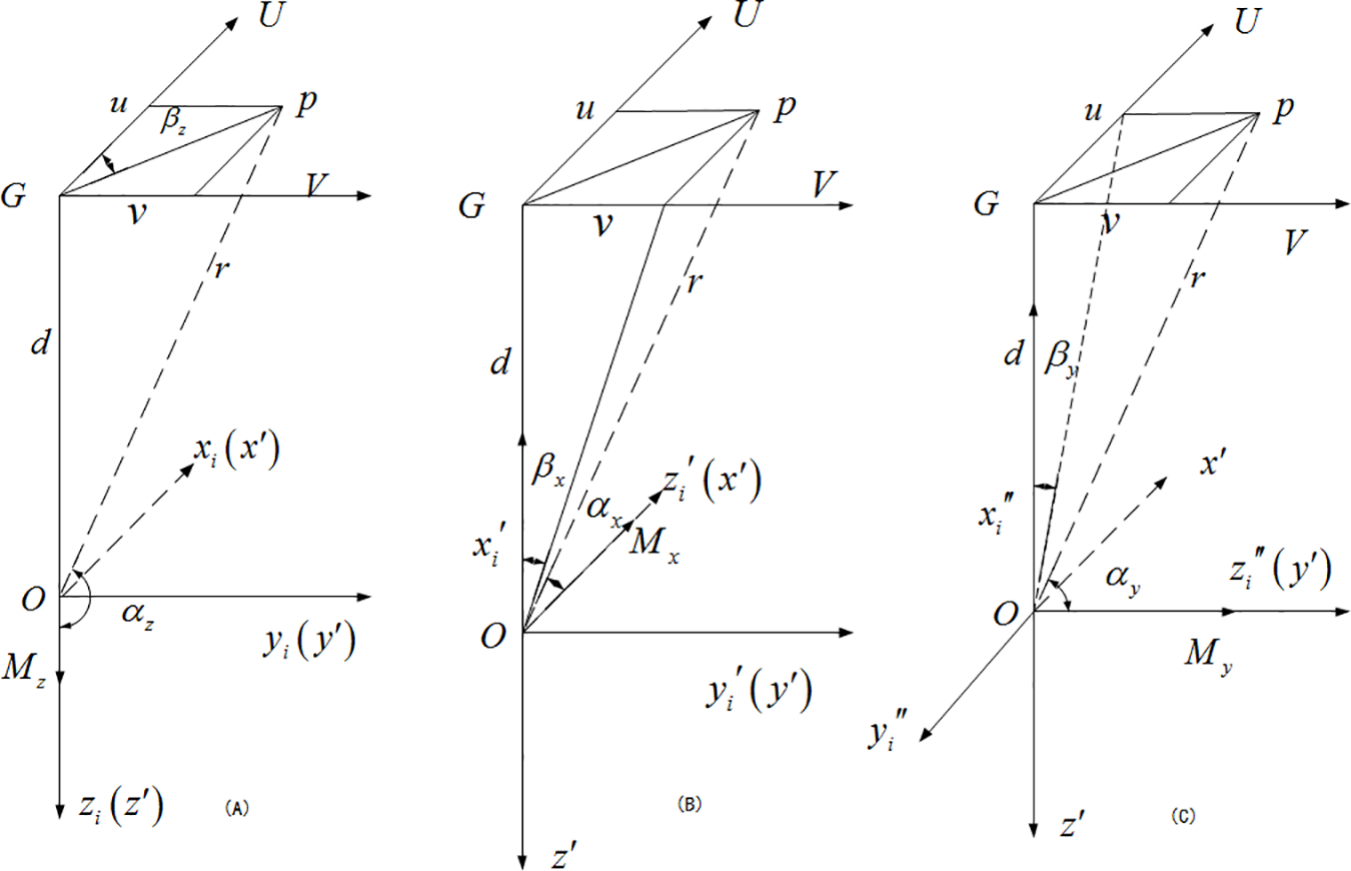
Magnetic field produced by induced magnetic moment at *p* point. (A) Induction field produced by magnetic moment *M*_*x*_ at point *p*. (B) Induction field produced by magnetic moment *M*_*y*_ at point *p*. (C) Induction field produced by magnetic moment *M*_*z*_ at point *p*.

At random attitude, the direction of the carrier’s induced magnetic moment aligns with the direction of the magnetic field and its three-axis direction coincides with the sensor’s three-axis direction. As shown in Fig 3(A), assume that the point *p* is the sensor’s position, *UGV* represents the rectangular coordinates of *p*’s plane, *og* is the vertical line between the *x*′*oy*′ and *ugv* planes, the *U*-axis is parallel to the *x*′-axis, and the *V*-axis is parallel to the *y*′-axis. As the sensor is installed in the carrier in a strap-down configuration, the relative positions of the coordinates *UGV* and the sensor’s coordinates *ox*′*y*′*z*′ remain unchanged. Meanwhile, spherical coordinates are created with the equivalent magnetic-moment point *o* as the center and the magnetized magnetic moment *M*_*z*_ as the reference to coincide with the *z*_*i*_-axis and *z*′-axis. Thus, the *M*_*z*_ direction is aligned with the *z*_*i*_ direction. Then, according to the magnetic-field theory of the magnetic dipole [14], we have

$$\{\begin{array}{l} \;F_{izx}=\frac{3\mu }{8\pi }\frac{M_{z}}{r^{3}}\sin2\alpha_{z}\cos\beta_{z};\\ \;F_{izy}=\frac{3\mu }{8\pi }\frac{M_{z}}{r^{3}}\sin2\alpha_{z}\sin\beta_{z};\\ F_{izz}=\frac{\mu }{2\pi }\frac{M_{z}}{r^{3}}\left(1-\frac{3}{2}\sin^{2}\alpha_{z}\right). \end{array},$$ (4)

where $r=\sqrt{u^{2}+v^{2}+d^{2}}\;\text{and}\;\alpha_{2}=\pi -\arctan\left(\frac{\sqrt{u^{2}+v^{2}}}{d}\right)\;\text{and}\;\beta_{2}=\arctan\left(\frac{v}{u}\right)$ and *μ* is the dielectric permeability, which is an inherent dielectric property.

With the magnetized magnetic moment *M*_*x*_ as the reference, keeping coordinates *UGV* and coordinates *ox*′*y*′*z*′ motionless and rotating coordinates *ox*_*i*_*y*_*i*_*z*_*i*_ along *y*_*i*_, we obtain coordinates $ox_{i}^{\prime }y_{i}^{\prime }z_{i}^{\prime }$, making the $z_{i}^{\prime }$-axis coincide with the *x*′-axis and the *M*_*x*_ direction align with the $z_{i}^{\prime }$ direction, as shown in Fig 3(B). Then,

$$\{\begin{array}{l} \;F_{ixx}=\frac{3\mu }{8\pi }\frac{M_{x}}{r^{3}}\sin2\alpha_{x}\cos\beta_{x};\\ \;F_{ixy}=\frac{3\mu }{8\pi }\frac{M_{x}}{r^{3}}\sin2\alpha_{x}\sin\beta_{x};\\ F_{ixz}=\frac{\mu }{2\pi }\frac{M_{x}}{r^{3}}\left(1-\frac{3}{2}\sin^{2}\alpha_{x}\right). \end{array},$$ (5)

where $\alpha_{x}=\arccos^{-1}\left(\frac{u}{r}\right)\;\text{and}\;\beta_{x}=\arctan\left(\frac{v}{d}\right)$,

With the magnetized magnetic moment *M*_*y*_ as the reference, keeping coordinates *UGV* and coordinates *ox*′*y*′*z*′ motionless and rotating coordinates *ox*_*i*_*y*_*i*_*z*_*i*_ along *x*_*i*_ on the basis of the previous rotation of coordinates *ox*_*i*_*y*_*i*_*z*_*i*_, we obtain $ox_{i}^{\prime \prime }y_{i}^{\prime \prime }z_{i}^{\prime \prime }$, making the $z_{i}^{\prime \prime }$-axis coincide with the *y*′-axis and the *M*_*y*_ direction align with the $z_{i}^{\prime \prime }$ direction, as shown in Fig 3(C). Then,

$$\{\begin{array}{l} \;F_{iyx}=\frac{3\mu }{8\pi }\frac{M_{y}}{r^{3}}\sin2\alpha_{y}\cos\beta_{y};\\ \;F_{iyy}=\frac{3\mu }{8\pi }\frac{M_{y}}{r^{3}}\sin2\alpha_{y}\sin\beta_{y};\\ F_{iyz}=\frac{\mu }{2\pi }\frac{M_{y}}{r^{3}}\left(1-\frac{3}{2}\sin^{2}\alpha_{y}\right). \end{array},$$ (6)

where $\alpha_{y}=\arccos^{-1}\left(\frac{v}{r}\right)\;\text{and}\;\beta_{y}=\arctan\left(\frac{u}{d}\right)$,

Then, at point *p*, the induced magnetic field *F*_*m*_ formed by the carrier’s soft magnetic material can be represented as

$$\{\begin{array}{c} F_{mx}=F_{ixz}-F_{iyy}+F_{izx};\\ F_{my}=F_{ixy}+F_{izy}+F_{iyz};\\ F_{mz}=-F_{ixx}-F_{iyx}+F_{izz}. \end{array},$$ (7)

Substituting Eqs (4), (5) and (6) into Eq (7) gives

$$\{\begin{array}{c} F_{mx}=b\frac{\mu }{2\pi }\frac{M_{x}}{r^{3}}\left(1-\frac{3}{2}\sin^{2}\alpha_{x}\right)-\frac{3\mu }{8\pi }\frac{M_{y}}{r^{3}}\sin2\alpha_{y}\sin\beta_{y}+\frac{3\mu }{8\pi }\frac{M_{z}}{r^{3}}\sin2\alpha_{z}\cos\beta_{z};\\ F_{my}=\frac{\mu }{2\pi }\frac{M_{y}}{r^{3}}\left(1-\frac{3}{2}\sin^{2}\alpha_{y}\right)+\frac{3\mu }{8\pi }\frac{M_{x}}{r^{3}}\sin2\alpha_{x}\sin\beta_{x}+\frac{3\mu }{8\pi }\frac{M_{z}}{r^{3}}\sin2\alpha_{z}\sin\beta_{z};\\ F_{mz}=\frac{\mu }{2\pi }\frac{M_{z}}{r^{3}}\left(1-\frac{3}{2}\sin^{2}\alpha_{z}\right)-\frac{3\mu }{8\pi }\frac{M_{x}}{r^{3}}\sin2\alpha_{x}\sin\beta_{x}-\frac{3\mu }{8\pi }\frac{M_{y}}{r^{3}}\sin2\alpha_{y}\sin\beta_{y}. \end{array},$$ (8)

Further reorganizing the above equation gives

$$\left[\begin{array}{c} F_{mx}\\ F_{my}\\ F_{mz} \end{array}\right]=\left[\begin{array}{ccc} \frac{\mu K_{x}}{2\pi r^{3}}\left(1-\frac{3}{2}\sin^{2}\alpha_{x}\right) & -\frac{3\mu K_{y}}{8\pi r^{3}}\sin2\alpha_{y}\sin\beta_{y} & \frac{3\mu K_{z}}{8\pi r^{3}}\sin2\alpha_{z}\cos\beta_{z}\\ \frac{3\mu K_{x}}{8\pi r^{3}}\sin2\alpha_{x}\sin\beta_{x} & \frac{\mu K_{y}}{2\pi r^{3}}\left(1-\frac{3}{2}\sin^{2}\alpha_{y}\right) & \frac{3\mu K_{z}}{8\pi r^{3}}\sin2\alpha_{z}\sin\beta_{z}\\ -\frac{3\mu K_{x}}{8\pi r^{3}}\sin2\alpha_{x}\cos\beta_{x} & -\frac{3\mu K_{y}}{8\pi r^{3}}\sin2\alpha_{y}\cos\beta_{y} & \frac{\mu K_{z}}{2\pi r^{3}}\left(1-\frac{3}{2}\sin^{2}\alpha_{z}\right) \end{array}\right]\left[\begin{array}{c} H_{ix}\\ H_{iy}\\ H_{iz} \end{array}\right],$$ (9)

where the coefficient matrix on the right contains 6 unknowns: *u*, *v*, *d*, *K*_*x*_, *K*_*y*_ and *K*_*2*._ These 6 unknowns are all constants for a carrier installed with a strap-down magnetic sensor. Eq (9) can be simplified to the following.

$$\left[\begin{array}{c} F_{mx}\\ F_{my}\\ F_{mz} \end{array}\right]=\left[\begin{array}{ccc} a_{xx} & a_{xy} & a_{xz}\\ a_{yx} & a_{yy} & a_{yz}\\ a_{zx} & a_{zy} & a_{zz} \end{array}\right]\left[\begin{array}{c} H_{ix}\\ H_{iy}\\ H_{iz} \end{array}\right].$$ (10)

The generation of random magnetic field contributes to the imbalanced variation of current inside the carrier and interference in the operation of onboard radio-transmission equipment. This random interfering field is not a major component of the carrier’s magnetic field and can be reduced or eliminated through a rational wiring plan. Under the condition of only considering the interference from the electromagnetic environment inside the carrier, the measured value of the geomagnetic-field vector in the carrier’s coordinates can be represented as follows:

$$H_{m}=A_{soft}\left(H_{i}+H_{hard}+H_{w}\right),$$ (11)

where matrix *A*_*soft*_ represents the interference generated by the soft magnetic material’s induction field on the measurement, and it is specifically expressed as follows:

$$A_{soft}=\left[\begin{array}{ccc} 1+a_{xx} & a_{xy} & a_{xz}\\ a_{yx} & 1+a_{yy} & a_{yz}\\ a_{zx} & a_{zy} & 1+a_{zz} \end{array}\right],$$

where *a*_*ij*_ is the interference coefficient of the induced magnetic field in which the *i* direction is acted on by the *j* direction; 1+*a*_*ij*_ represents the increased multiple of strength of the magnetic field in the magnetic sensor’s *i*-axis direction under the influence of the induced magnetic field; *H*_*hard*_ represents the hard magnetic interference and is specifically expressed as *H*_*hard*_ = [*H*_*hardx*_ *H*_*hardy*_ *H*_*hardz*_]^T^; and *H*_*w*_ represents the random magnetic field inside the carrier. Eq (11) shows the interference model of the external electromagnetic environment that is generally adopted. This model only considers the electromagnetic-interference field inside the carrier and neglects the geomagnetic daily variation field, which has significant influence on geomagnetic-field measurement. It was noted that the geomagnetic daily variation field is one of the important sources of error for geomagnetic-field measurement and has significant influence on geomagnetic navigation [15]. The geomagnetic daily variation field component can be divided into two parts: the regular variation field and the irregular variation field. The regular variation field includes daily variations and annual variations, and its variation is continuous and independent of time; the irregular variation field includes magnetic storm and substorm, and its variation is sporadic and discontinuous. The regular variation field’s interference imposed on the magnetic field can be regarded as a fixed vector. The irregular variation field is regarded as noise in this paper. Therefore, by comprehensively considering the influence of the magnetic sensor’s external electromagnetic environment on magnetic-field measurement, Eq (11) can be written as follows.

$$H_{m}=A_{soft}\left(H_{i}+H_{hard}+H_{w}+H_{r}+H_{ir}\right),$$ (12)

where *H*_*r*_ represents the regular variation field and *H*_*ir*_ the irregular variation field in the geomagnetic daily variation field.

### 2.4. Measurement-error model

In summary, comprehensively considering the magnetic sensor’s manufacturing error, installation error and the error of the magnetic sensor’s external magnetic interference can produce the magnetic sensor’s geomagnetic-field measurement-error model:

$$H_{m}=SE_{p}A_{soft}\left(H_{i}+H_{hard}+H_{w}+H_{r}+H_{ir}\right)+b_{o}=CH_{i}+b+\varepsilon ,$$ (13)

Where *C* = *SE*_*p*_*A*_*soft*_; *b* = *SE*_*p*_*A*_*soft*_(*H*_*hard*_ + *H*_*o*_) + *b*_*o*_ represents the offset of the central coordinates of the geomagnetic-field track, described as *b* = [*b*_*x*_ *b*_*y*_ *b*_*z*_]^T^; and *ε* = *SE*_*p*_*A*_*soft*_(*H*_*w*_ + *H*_*ir*_) represents the magnetic sensor’s measuring noise.

Normally, *C* is an invertible matrix and the noise *ε* can be eliminated with the use of a certain device and technology [4], thus the magnetic sensor’s measurement-error compensation model can be described as

$$H_{i}=C^{-1}\left(H_{m}-b\right),$$ (14)

where the inverse of *C* can be described as $C^{-1}=\left[\begin{array}{ccc} c_{xx} & c_{xy} & c_{xz}\\ c_{yx} & c_{yy} & c_{yz}\\ c_{zx} & c_{zy} & c_{zz} \end{array}\right]$.

## 3. Filter design

It has been previously noted that the sensitivity-error matrix *S*, installation-error matrix *E*_*p*_, hard magnetic interference *H*_*hard*_ and regular variation field *H*_*r*_ in the geomagnetic daily variation field can be regarded as fixed values for handling. However, only when the aircraft’s attitude and geomagnetic field remain unchanged will the interference-error matrix *A*_*soft*_ be fixed. Therefore, the equation does not satisfy the linearity condition.

In designing the filter, choose all elements in the matrix *C* and vector *b*’s three components as the system’s state variables, totaling 12 dimensions:

$$x={\left[c_{xx}\;c_{xy}\;c_{xz}\;c_{yx}\;c_{yy}\;c_{yz}\;c_{zx}\;c_{zy}\;c_{zz}\;b_{x}\;b_{y}\;b_{z}\right]}^{T},$$ (15)

As the parameters in the state variables are constant values, the filter system’s state equation can be expressed as

$$\dot{x}\left(t\right)=w\left(t\right),$$ (16)

where *w*(*t*) is process noise. Assume that this noise is zero-mean Gaussian noise.

Normally there are two options for the selection of the observable, namely the strength and vector of the geomagnetic-field. The algorithm is much simpler when the strength of the geomagnetic-field is used as the observable; therefore, this paper uses the total strength of the geomagnetic-field, including the noise measured using a magnetic sensor as the observable:

$$z=H_{m}{}^{\text{T}}H_{m},$$ (17)

Further reorganizing Eq (14) gives the following equation.

$$\begin{array}{l} H_{i}{}^{\text{T}}H_{i}=H_{m}{}^{\text{T}}{\left(C^{-1}\right)}^{\text{T}}\left(C^{-1}\right)H_{m}-2b^{\text{T}}{\left(C^{-1}\right)}^{\text{T}}\left(C^{-1}\right)H_{m}+b^{\text{T}}b\\ \;-2\varepsilon^{\text{T}}{\left(C^{-1}\right)}^{\text{T}}\left(C^{-1}\right)H_{m}+2b^{\text{T}}{\left(C^{-1}\right)}^{\text{T}}\left(C^{-1}\right)\varepsilon +\varepsilon^{\text{T}}\varepsilon \end{array},$$ (18)

Eq (18) can be transformed into the following equation.

$$\begin{array}{l} H_{i}{}^{\text{T}}H_{i}=H_{m}{}^{\text{T}}H_{m}+H_{m}{}^{\text{T}}(T-I)H_{m}-2b^{\text{T}}TH_{m}+b^{\text{T}}b\\ \;-2\varepsilon^{\text{T}}TH_{m}+2b^{\text{T}}T\varepsilon +\varepsilon^{\text{T}}\varepsilon \end{array},$$ (19)

where *T* = (*C*^−1^)^T^ (*C*^−1^). From Eq (19), we obtain

$$H_{m}{}^{\text{T}}H_{m}=H_{i}{}^{\text{T}}H_{i}-H_{m}{}^{\text{T}}(T-I)H_{m}+2b^{\text{T}}TH_{m}-b^{\text{T}}b+\nu ,$$ (20)

where *ν* = 2*ε*^T^*TH*_*m*_−2*b*^T^*Tε*−*ε*^T^*ε* is regarded as process noise. Assume it is zero-mean Gaussian noise. Combining Eqs (17) and (20), we obtain the filter’s observation equation as follows.

$$z=H_{i}{}^{\text{T}}H_{i}-H_{m}{}^{\text{T}}(T-I)H_{m}+2b^{\text{T}}TH_{m}-b^{\text{T}}b+\nu ,$$ (21)

The above equation can be further specified as follows.

$$\begin{array}{l} z=H_{ix}^{2}+H_{iy}^{2}+H_{iz}^{2}-\left(c_{xx}^{2}+c_{yx}^{2}+c_{zx}^{2}-1\right)H_{mx}^{2}-\left(c_{xy}^{2}+c_{yy}^{2}+c_{zy}^{2}-1\right)H_{my}^{2}\\ \;-\left(c_{xz}^{2}+c_{yz}^{2}+c_{zz}^{2}-1\right)H_{mz}^{2}-2\left(c_{xx}c_{xy}+c_{yx}c_{yy}+c_{zx}c_{zy}\right)H_{mx}H_{my}\\ \;-2\left(c_{xx}c_{xz}+c_{yx}c_{yz}+c_{zx}c_{zz}\right)H_{mx}H_{mz}-2\left(c_{xy}c_{xz}+c_{yy}c_{yz}+c_{zy}c_{zz}\right)H_{my}H_{mz}\\ \;+2\left(c_{xx}^{2}+c_{yx}^{2}+c_{zx}^{2}+c_{xx}c_{xy}+c_{yx}c_{yy}+c_{zx}c_{zy}+c_{xx}c_{xz}+c_{yx}c_{yz}+c_{zx}c_{zz}\right)b_{x}H_{mx}\\ \;+2\left(c_{xx}c_{xy}+c_{yx}c_{yy}+c_{zx}c_{zy}+c_{xy}^{2}+c_{yy}^{2}+c_{zy}^{2}+c_{xx}c_{xz}+c_{yx}c_{yz}+c_{zx}c_{zz}\right)b_{y}H_{my}\\ \;+2\left(c_{xx}c_{xz}+c_{yx}c_{yz}+c_{zx}c_{zz}+c_{xx}c_{xz}+c_{yx}c_{yz}+c_{zx}c_{zz}+c_{xz}^{2}+c_{yz}^{2}+c_{zz}^{2}\right)b_{z}H_{mz}\\ \;-b_{x}^{2}-b_{y}^{2}-b_{z}^{2}+\nu \end{array},$$ (22)

Eqs (16) and (21) constitute the filter mathematical model estimated using the magnetic sensor’s error parameters. As the observation equation does not satisfy the linearity condition, it is not possible to directly perform parameter estimation using the Kalman-filter algorithm [21]. This paper used an extended Kalman-filter algorithm to perform parameter estimation. Based on Eqs (16) and (21), the filter model can be expressed as follows.

$$\{\begin{array}{l} \;\dot{x}\left(t\right)=w\left(t\right)\\ z\left(t\right)=f\left(x\left(t\right),t\right)+\nu \left(t\right) \end{array},$$ (23)

where *f*( ) represents the non-linear function of *x*(*t*). The system model is linearized to as follows.

$$\{\begin{array}{c} \delta x_{k}=\Phi_{k,k-1}\delta x_{k-1}+w_{k-1}\\ \delta z_{k}=H_{k}\delta x_{k}+\nu_{k} \end{array},$$ (24)

where the state-transition matrix Φ_*k*,*k*−1_ is a 12-dimensional unit matrix and the observation matrix *H*_*k*_ is the Jacobi matrix of partial derivatives of with respect to the state variable *x*(*t*).

$$H_{k}=\frac{\partial f\left(x\left(t\right),t\right)}{\partial x\left(t\right)}=\left[\frac{\partial f}{\partial c_{xx}},\frac{\partial f}{\partial c_{xy}},\frac{\partial f}{\partial c_{xz}},\frac{\partial f}{\partial c_{yx}},\frac{\partial f}{\partial c_{yy}},\frac{\partial f}{\partial c_{yz}},\frac{\partial f}{\partial c_{zx}},\frac{\partial f}{\partial c_{zy}},\frac{\partial f}{\partial c_{zz}},\frac{\partial f}{\partial b_{x}},\frac{\partial f}{\partial b_{y}}\frac{\partial f}{\partial b_{z}}\right],$$ (25)

where

$$\{\begin{array}{c} \frac{\partial f}{\partial c_{xx}}=2\left(-c_{xx}H_{mx}^{2}-c_{xy}H_{mx}H_{my}-c_{xz}H_{mx}H_{mz}\right)+2c_{xy}b_{y}H_{my}\\ +2c_{xz}b_{z}H_{mz}+2\left(c_{xx}+c_{xy}+c_{xz}\right)b_{x}H_{mx}\\ \frac{\partial f}{\partial c_{xy}}=2\left(-c_{xy}H_{my}^{2}-c_{xx}H_{mx}H_{my}-c_{xz}H_{my}H_{mz}\right)+2c_{xx}b_{x}H_{mx}\\ +2c_{xz}b_{z}H_{mz}+2\left(c_{xx}+c_{xy}+c_{xz}\right)b_{y}H_{my}\\ \frac{\partial f}{\partial c_{xz}}=2\left(-c_{xz}H_{mz}^{2}-c_{xx}H_{mx}H_{mz}-c_{xy}H_{my}H_{mz}\right)+2c_{xx}b_{x}H_{mx}\\ +2c_{xy}b_{y}H_{my}+2\left(c_{xx}+c_{xy}+c_{xz}\right)b_{z}H_{mz}\\ \frac{\partial f}{\partial c_{yx}}=2\left(-c_{yx}H_{mx}^{2}-c_{yy}H_{mx}H_{my}-c_{yz}H_{mx}H_{mz}\right)+2c_{yy}b_{y}H_{my}\\ +2c_{yz}b_{z}H_{mz}+2\left(c_{yx}+c_{yy}+c_{yz}\right)b_{x}H_{mx}\\ \frac{\partial f}{\partial c_{yy}}=2\left(-c_{yy}H_{my}^{2}-c_{yx}H_{mx}H_{my}-c_{yz}H_{my}H_{mz}\right)+2c_{yx}b_{x}H_{mx}\\ +2c_{yz}b_{z}H_{mz}+2\left(c_{yx}+c_{yy}+c_{yz}\right)b_{y}H_{my}\\ \frac{\partial f}{\partial c_{yz}}=2\left(-c_{yz}H_{mz}^{2}-c_{yx}H_{mx}H_{mz}-c_{yy}H_{my}H_{mz}\right)+2c_{yx}b_{x}H_{mx}\\ +2c_{yy}b_{y}H_{my}+2\left(c_{yx}+c_{yy}+c_{yz}\right)b_{z}H_{mz}\\ \frac{\partial f}{\partial c_{zx}}=2\left(-c_{zx}H_{mx}^{2}-c_{zy}H_{mx}H_{my}-c_{zz}H_{mx}H_{mz}\right)+2c_{zy}b_{y}H_{my}\\ +2c_{zz}b_{z}H_{mz}+2\left(c_{zx}+c_{zy}+c_{zz}\right)b_{x}H_{mx}\\ \frac{\partial f}{\partial c_{zy}}=2\left(-c_{zy}H_{my}^{2}-c_{zx}H_{mx}H_{my}-c_{zz}H_{my}H_{mz}\right)+2c_{zx}b_{x}H_{mx}\\ +2c_{zz}b_{z}H_{mz}+2\left(c_{zx}+c_{zy}+c_{zz}\right)b_{y}H_{my}\\ \frac{\partial f}{\partial c_{zz}}=2\left(-c_{zz}H_{mz}^{2}-c_{zx}H_{mx}H_{mz}-c_{zy}H_{my}H_{mz}\right)+2c_{zx}b_{x}H_{mx}\\ +2c_{zy}b_{y}H_{my}+2\left(c_{zx}+c_{zy}+c_{zz}\right)b_{z}H_{mz}\\ \frac{\partial f}{\partial b_{x}}=2\left(c_{xx}^{2}+c_{yx}^{2}+c_{zx}^{2}+c_{xx}c_{xy}+c_{yx}c_{yy}+c_{zx}c_{zy}+c_{xx}c_{xz}+c_{yx}c_{yz}+c_{zx}c_{zz}\right)H_{mx}-2b_{x}\\ \frac{\partial f}{\partial b_{x}}=2\left(c_{xx}c_{xy}+c_{yx}c_{yy}+c_{zx}c_{zy}+c_{xy}^{2}+c_{yy}^{2}+c_{zy}^{2}+c_{xy}c_{xz}+c_{yy}c_{yz}+c_{zy}c_{zz}\right)H_{my}-2b_{y}\\ \frac{\partial f}{\partial b_{z}}=2\left(c_{xx}c_{xz}+c_{yx}c_{yz}+c_{zx}c_{zz}+c_{xy}c_{xz}+c_{yy}c_{yz}+c_{zy}c_{zz}+c_{xz}^{2}+c_{yz}^{2}+c_{zz}^{2}\right)H_{mz}-2b_{z} \end{array}.$$

In summary, the parameter-estimation algorithm of the magnetic sensor’s measurement-error model based on an extended Kalman-filter structure is as follows:

$$\{\begin{array}{c} {\overset{⌢}{x}}_{k,k-1}=\Phi_{k,k-1}{\overset{⌢}{x}}_{k-1}\\ P_{k,k-1}=P_{k-1}+Q_{k-1}\\ K_{k}=P_{k,k-1}H_{k}{}^{T}{\left(H_{k}P_{k,k-1}H_{k}{}^{T}+R_{k}\right)}^{-1}\\ {\overset{⌢}{x}}_{k}={\overset{⌢}{x}}_{k,k-1}+K_{k}\left(z_{k}-f\left({\overset{⌢}{x}}_{k,k-1},k\right)\right)\\ P_{k}=\left(I-K_{k}H_{k}\right)P_{k,k-1}{\left(I-K_{k}H_{k}\right)}^{\text{T}}+K_{k}R_{k}{\left(K_{k}\right)}^{\text{T}} \end{array},$$ (26)

The model’s error parameter can be estimated by the above filtering process, and the compensation of magnetic measurement data can thus be made according to Eq (14).

## 4. Experimental verification

### 4.1. Simulation and analysis

This paper adopts a numerical simulation to verify the validity of the compensation algorithm. First, assuming the strength of the clean geomagnetic-field of the location to be 45306nT, 1,000 groups of data of the geomagnetic-field under ideal conditions were generated; second, the author further generated output data, including interfering items in the light of the conditions set in Table 1, according to an already-built error model as observables for filter estimation. On the basis of the previous two steps, the author performed an estimation of the error model’s parameters with the designed filter. After obtaining the estimated values of the parameters, the author compensated the existing parameters and examined the result of the compensation. In the meantime, the author used the RLS (recursive least-square) method and ellipsoid-fitting method to compensate the actual output data and compared the effects of the three compensation methods. The parameters’ initial states in the three compensation methods were the same.

**Table 1:** Parameters of magnetic measurement-error model.

Axial direction	*b*	Measurement-noise standard deviation	Error-coefficient matrix *C*
X axis	600/nT	200/nT	$C=\left[\begin{array}{ccc} 1.362 & 0.002 & 0.001\\ 0.001 & 0.861 & 0.002\\ 0.002 & 0.001 & 1.046 \end{array}\right]$
Y axis	700/nT	200/nT	
Z axis	750/nT	200/nT	

#### 4.1.1. Condition settings of simulation

Settings of the relative values, including the error-coefficient matrix *C*, are as shown in Table 1.

To test the filter ability, take into account some errors in initial states during the setting of initial state vector, and set the initial state vector as:

$$x_{0}={\left[1.5,0.05,0.05,\;0.05,0.5,0.05,\;0.05,0.05,1,\;0,\;0,\;0\right]}^{\text{T}}.$$

In general, the measuring noise covariance matrix can be obtained by means of some offline samples, and it represents the measuring noise level of sensor; the process noise covariance matrix is used to describe the uncertainty of model. According to experience, the performance and convergence of estimation algorithm are subject to the impact of parameter values in the covariance matrix; thus it's necessary to conduct some simulations to determine the parameter values. In view of the fact that the values of all principal diagonal elements of error-coefficient matrix ***C*** are all around 1 and those of other elements close to 0, and the values of three elements of vector ***b*** are relatively large, the initial covariance matrix and the process noise covariance matrix are respectively set as:

$$P_{0}=\text{diag}\left(\left[1;1^{-4};1^{-4};1^{-4};1;1^{-4};1^{-4};1^{-4};1;10^{4};10^{4};10^{4}\right]\right);$$

$$\text{Q}=\text{diag}([10^{-4}{;10}^{-4}{;10}^{-4}{;\;10}^{-4}{;10}^{-4}{;10}^{-4}{;\;10}^{-4}{;10}^{-4}{;10}^{-4}{;\;10}^{4}{;10}^{4}{;10}^{4}]).$$

Since the standard deviation of measuring noise is set as 100, the initial value of observation noise covariance matrix is set as:

$$R=\text{diag}\left(\left[10^{4};10^{4};10^{4}\right]\right).$$

#### 4.1.2. Results of comparison with RLS method

**Fig 4:**
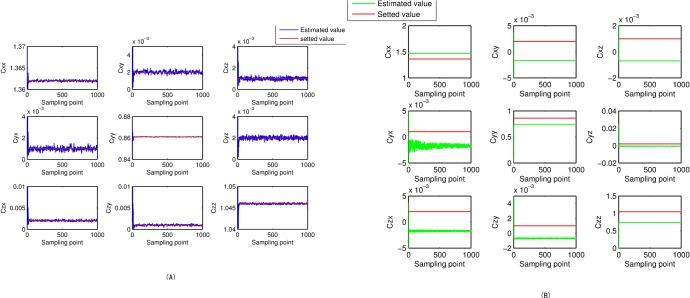
Estimation results of matrix *C*’s parameters. (A) Extended Kalman filter. (B) Recursive least square.

**Fig 5:**
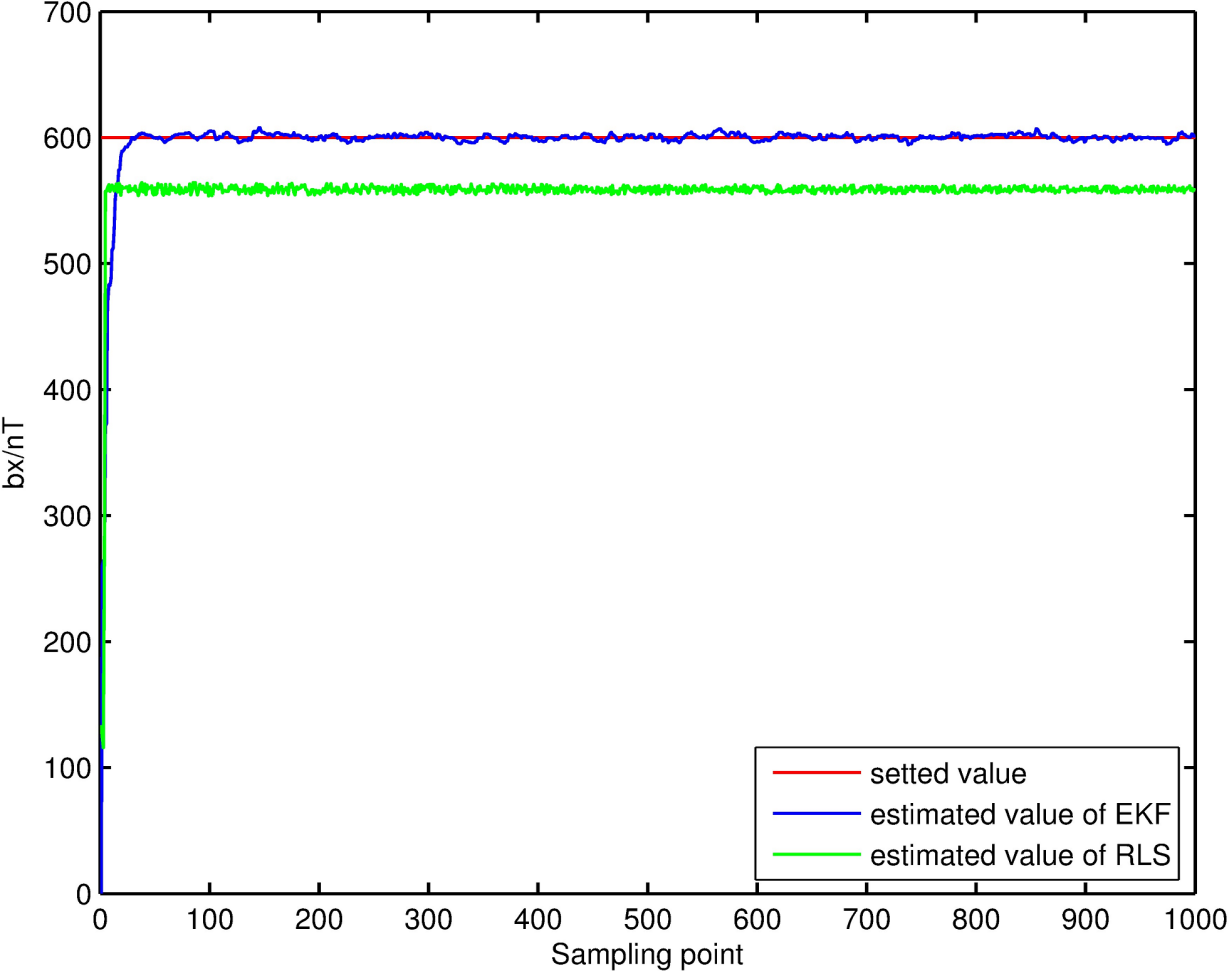
Estimation result of the *b*_*x*_ filter.

**Fig 6:**
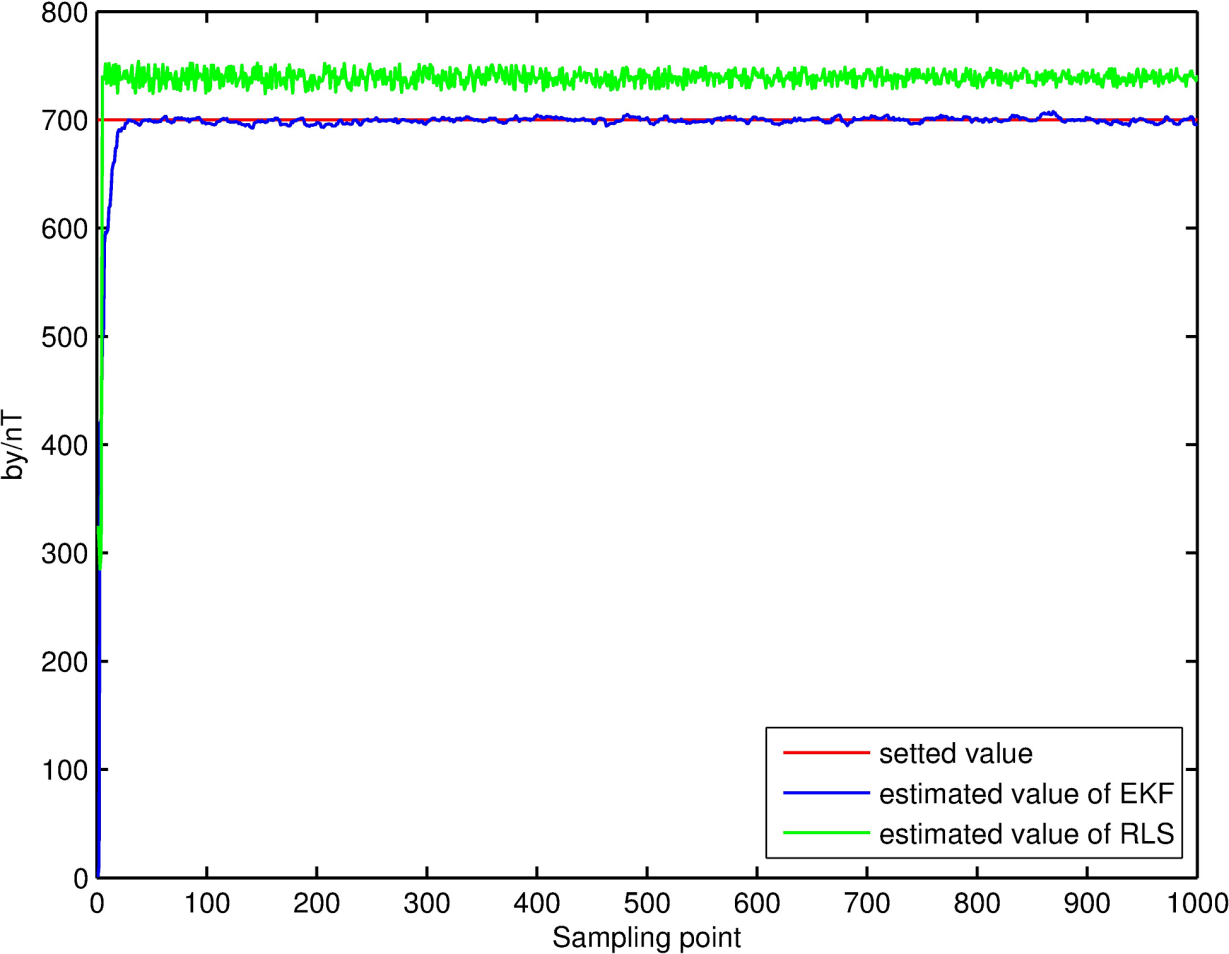
Estimation result of the *b*_*y*_ filter.

**Fig 7:**
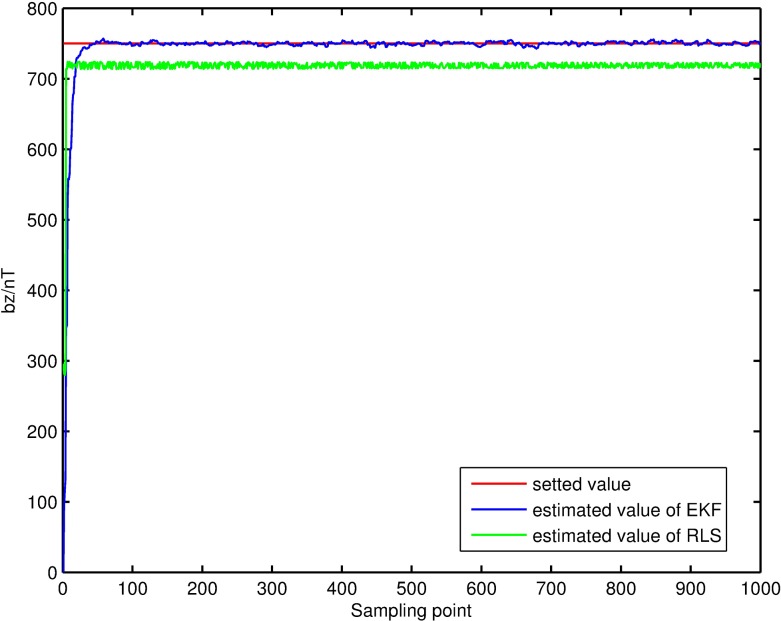
Estimation result of the *b*_*z*_ filter.

Filtering result of parameters of magnetic measurement-error model. The filtering results, shown in Figs 4–7, demonstrate that the parameter estimations achieve good convergence. Fig 4 shows that the accuracy of the extended Kalman-filter estimation is higher and the parameter estimation finally converges to a set value, and that the parameter estimation using the RLS method exhibits a certain deviation. Figs 5–7 show that the estimation result of *b*_*x*_,*b*_*y*_,*b*_*z*_ under the extended Kalman filter is ideal and has good convergence to the values 601.44nT, 695.63nT and 748.50nT when filtering finishes. Its deviations from the set values are 1.44nT, 4.47nT and 1.50nT, respectively, and the estimated error is within 0.64%. The final estimation results of the RLS method are 558.48nT, 740.98nT and 715.65nT. Its deviations from the set values are 41.52nT, 40.98nT and 35.35nT, respectively, and the estimated error is within 6.92%.

**Fig 8:**
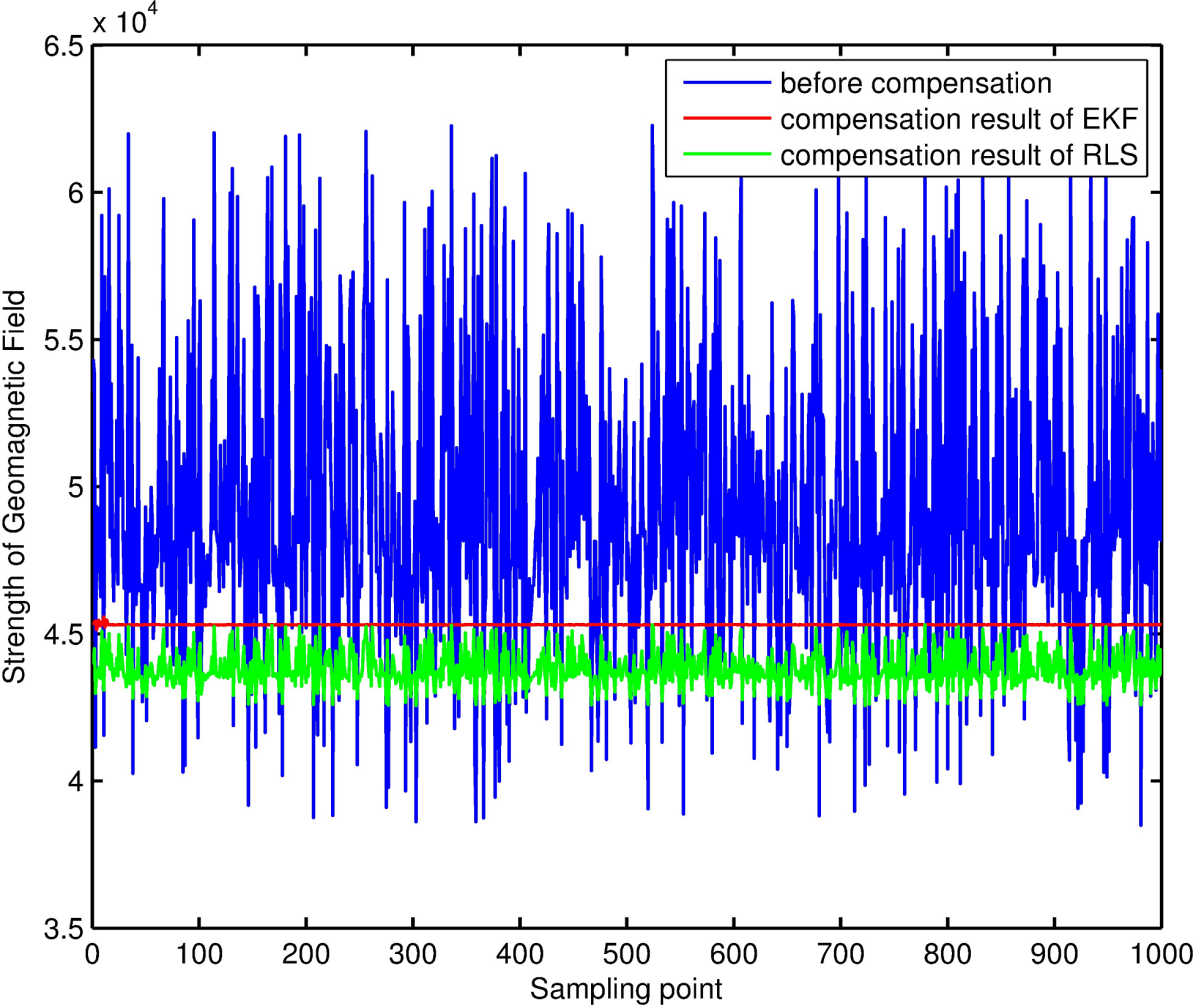
Strength of geomagnetic field before & after compensation.

Comparison of strengths of geomagnetic field before and after compensation. After obtaining the estimation results of filtering, the result of compensating magnetic-measurement data is as shown in Fig 8: the strength of the geomagnetic-field fluctuates greatly before compensation and the fluctuation is obviously reduced after compensation using the extended Kalman-filter method and the RLS method. However, the former’s compensation effect is much better, as the fluctuation of the strength of the geomagnetic-field basically disappears and remains close to the real value after being compensated by the former. Although the fluctuation of the strength of the geomagnetic-field is obviously reduced after being compensated by the latter, its fluctuation margin is larger than that of the former and deviates from the real value. It is known from calculation that the compensation error of the extended Kalman-filter can mostly remain within 5nT, with a mean value of 0.49nT and standard deviation of 16.23nT; however, the mean value of the compensation error of RLS is -297.99nT and the standard deviation is 611.90nT.

#### 4.1.3. Results of comparison with ellipsoid fitting algorithm

**Fig 9:**
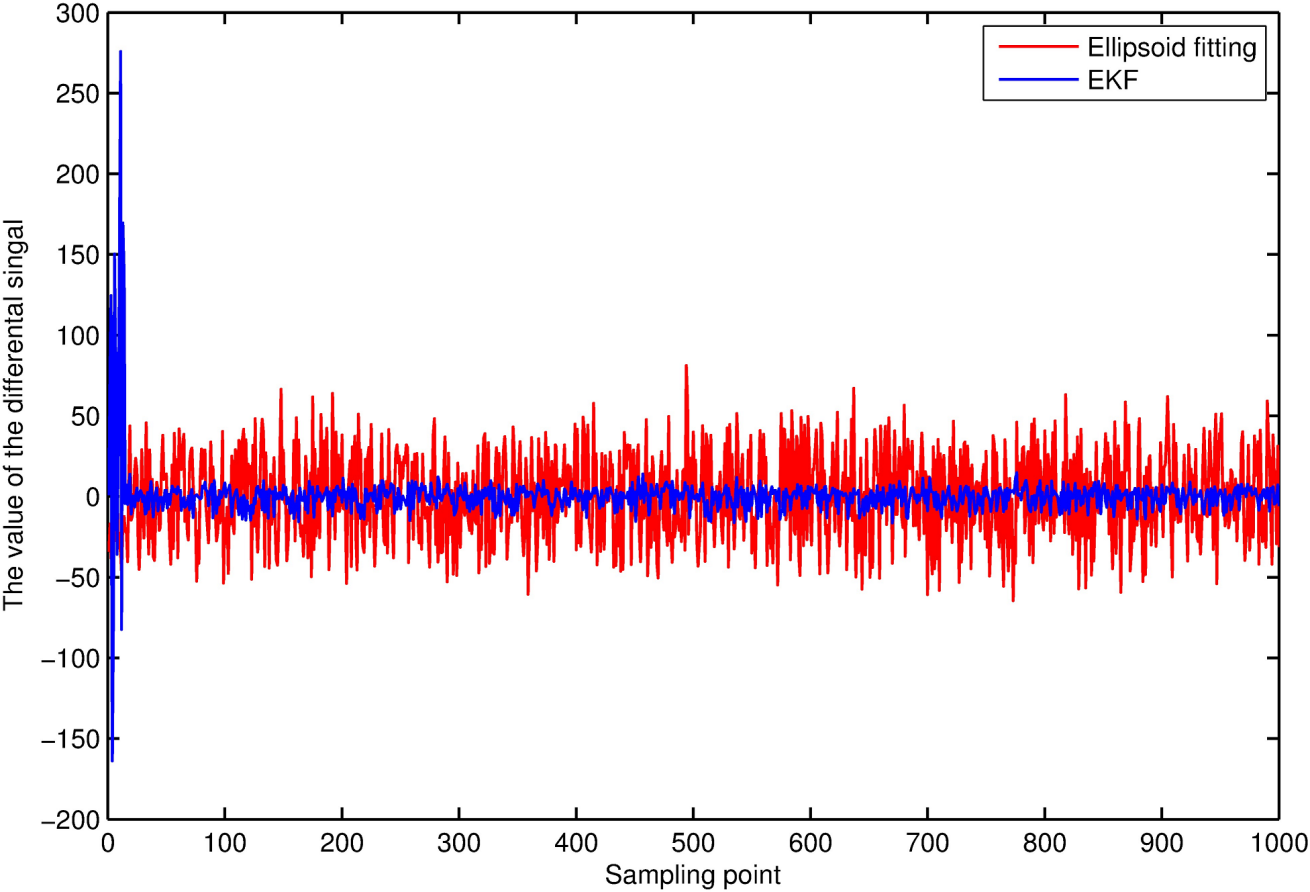
Differential value before & after the signal compensation.

The least-square method based on the ellipsoid constraint is used for comparison with the method proposed in the paper. The ellipsoid fitting method is unable to directly work out the parameters of model, so the paper only lists the comparison diagram of geomagnetic field's differential signals after compensations with the two methods as shown in Fig 9.

It is known from calculation that the compensation error of the extended Kalman-filter can mostly remain within 5nT, with a mean value of 0.49nT and standard deviation of 16.23nT; however, the mean value of the compensation error of ellipsoid fitting is -0.37nT and the standard deviation is 27.63nT. The method proposed in the paper achieves better compensation effect than the ellipsoid fitting algorithm.

### 4.2. Experiment and analysis

**Fig 10:**
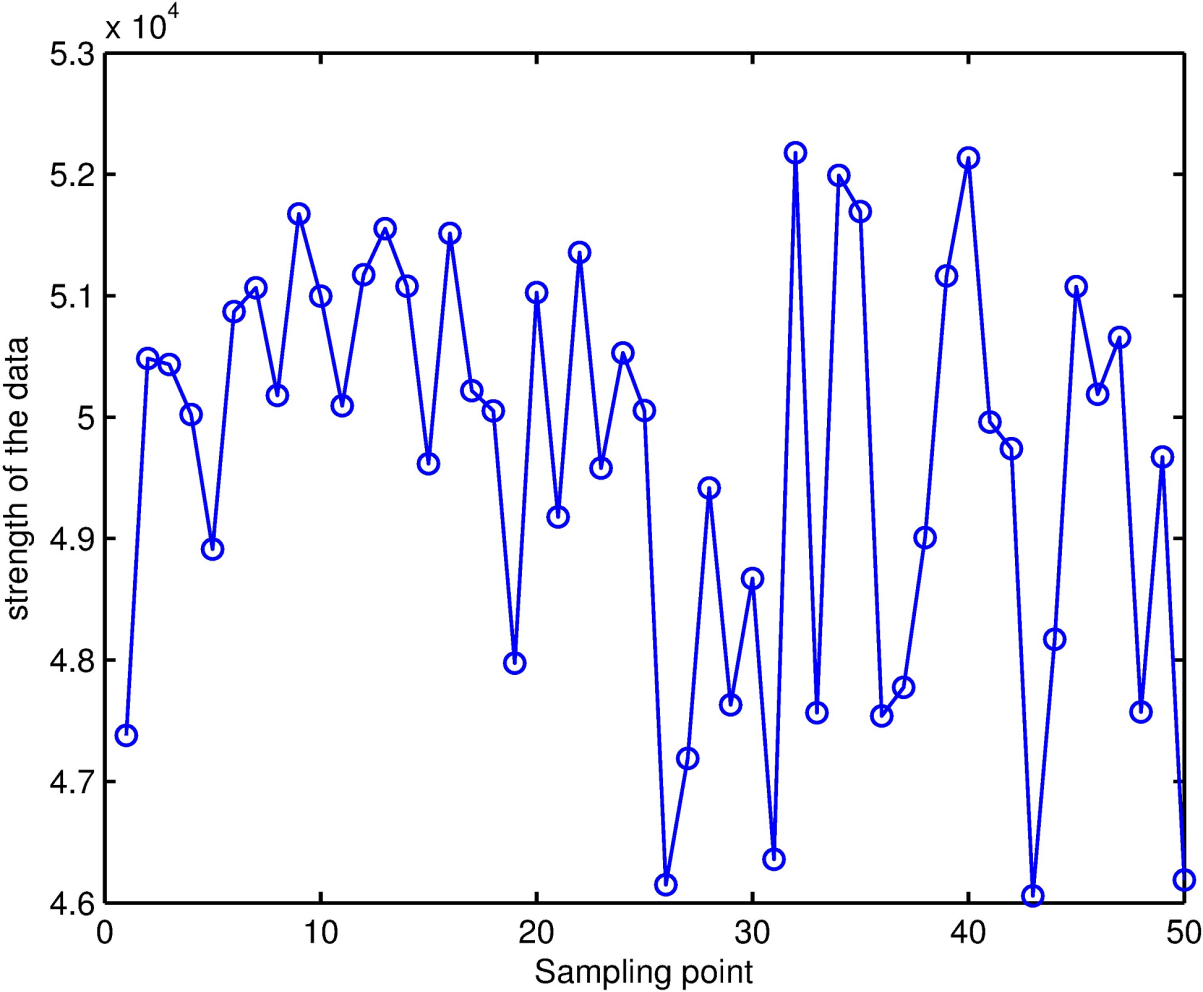
Strength of the geomagnetic field of collected data.

**Fig 11:**
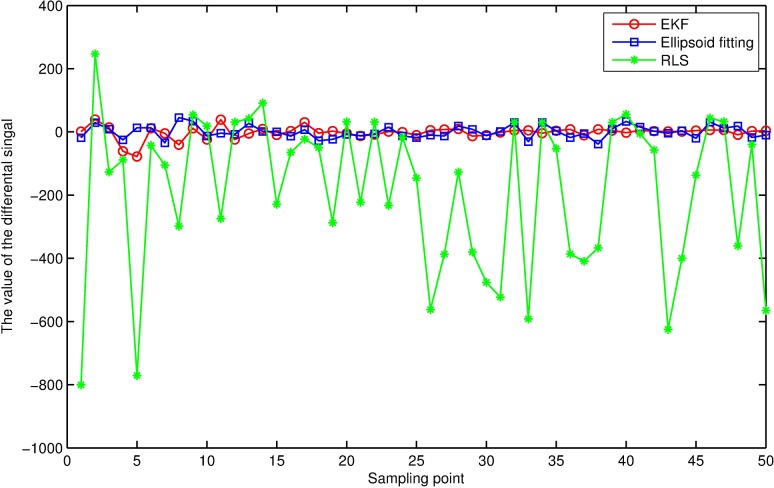
Differential value before & after the signal compensation.

To examine the effect of the method proposed in this paper, an experiment was conducted. First, a clean place with respect to the electromagnetic environment was selected and the strength of the clean geomagnetic field of this place was measured as 48476nT. Second, a three-axis sensor and ferromagnetic materials on a three-axis non-magnetic rotary table were fixed, and the non-magnetic rotary table was randomly rotated in space. The sensor’s output data was recorded and 50 groups of data were collected. The strength of the data is as shown in Fig 10, and compensation error is as shown in Fig 11. It is known from calculation that the mean value of the compensation error of the extended Kalman filter is -1.96nT and the standard deviation is 19.45nT; however, the mean value of the compensation error of RLS is -189.39nT and the standard deviation is 245.55nT. The mean value of the compensation error of ellipsoid fitting is -0.37nT and the standard deviation is 27.63nT. It's thus clear that the method proposed in the paper is obviously better than the least-square method with respect to compensation effect and meanwhile avoids the problem of matrix singularity likely to arise in the solving process of the latter. The method proposed in the paper achieves better compensation effect than the ellipsoid fitting algorithm and also avoids the problems, such as heavy computation and the need for good initial conditions, in the solving process of the latter.

## 5. Conclusion

The measurement of the geomagnetic field using a magnetic sensor is affected by multiple factors. To address these factors, this paper built a brand-new geomagnetic-measurement model that can accurately describe the source of measurement error. It not only considered the magnetic sensor’s manufacturing error, installation error and the interference from the external electromagnetic environment but also introduced the previously neglected geomagnetic daily variation field. The author designed an extended Kalman-filter according to the characteristics of the newly built model and performed experiment. The experimental results showed that parameter estimation with this method has good convergence, and that the strength of the geomagnetic-field after being compensated always remains close to the real value. Thus, this method is able to meet the demands of geomagnetic navigation for high accuracy of geomagnetic-field measurement. This compensation method has strong applicability due to its easy data collection and its ability to get rid of the dependence on high-precision measurement instruments.
